# Adult auditory brain responses to nestling begging calls in seasonal songbirds: an fMRI study in non-parenting male and female starlings (*Sturnus vulgaris*)

**DOI:** 10.3389/fnbeh.2024.1418577

**Published:** 2024-09-17

**Authors:** Nicholas Vidas-Guscic, Elisabeth Jonckers, Johan Van Audekerke, Jasmien Orije, Julie Hamaide, Gaurav Majumdar, Laurence Henry, Martine Hausberger, Marleen Verhoye, Annemie Van der Linden

**Affiliations:** ^1^Bio-Imaging Lab, Faculty of Pharmaceutical, Biomedical and Veterinary Sciences, University of Antwerp, Wilrijk, Antwerp, Belgium; ^2^µNEURO Research Centre of Excellence, University of Antwerp, Antwerp, Belgium; ^3^Université de Rennes, UMR 6552, Ethologie Animale et Humaine (EthoS), CNRS, Brittany, France; ^4^CNRS, UMR 8002, Centre de Neuroscience et de Cognition Intégrative (INCC), Université de Paris-Cité, Paris, France

**Keywords:** European starling, begging calls, caudomedial nidopallium, auditory perception, functional magnetic resonance imaging (fMRI), lateralization, songbird, neuroethology

## Abstract

The present study aims to investigate whether begging calls elicit specific auditory responses in non-parenting birds, whether these responses are influenced by the hormonal status of the bird, and whether they reflect biparental care for offspring in the European starling (*Sturnus vulgaris*). An fMRI experiment was conducted to expose non-parenting male and female European starlings to recordings of conspecific nestling begging calls during both artificially induced breeding and non-breeding seasons. This response was compared with their reaction to conspecific individual warbling song motifs and artificial pure tones, serving as social species-specific and artificial control stimuli, respectively. Our findings reveal that begging calls evoke a response in non-parenting male and female starlings, with significantly higher responsiveness observed in the right Field L and the Caudomedial Nidopallium (NCM), regardless of season or sex. Moreover, a significant seasonal variation in auditory brain responses was elicited in both sexes exclusively by begging calls, not by the applied control stimuli, within a ventral midsagittal region of NCM. This heightened response to begging calls, even in non-parenting birds, in the right primary auditory system (Field L), and the photoperiod induced hormonal neuromodulation of auditory responses to offspring’s begging calls in the secondary auditory system (NCM), bears resemblance to mammalian responses to hunger calls. This suggests a convergent evolution aimed at facilitating swift adult responses to such calls crucial for offspring survival.

## Introduction

Seasonal breeding in birds is regulated by photoperiod, which is the duration of daylight within a 24-h period. This natural cue serves as an accurate indicator of the approaching spring and summer, especially for birds living in higher latitudes (Gwinner, 2003). Changes in photoperiod are detected by endogenous control mechanisms that regulate seasonal processes, such as reproduction. Increasing day length (or photostimulation) during spring stimulates the secretion of gonadotropin-releasing hormone (GnRH) and subsequent gonadal maturation in preparation for breeding (Yoshimura et al., 2003). Breeding is timed to coincide with periods of food abundance (Daan et al., 1989) and therefore, prolonged exposure to extended daylight during the summer triggers a state called photorefractoriness. This condition leads to a significant decline in hypothalamic GnRH, causing the gonads to regress and revert to a pre-pubertal state. In autumn, exposure to short photoperiods, when day length falls below approximately 11.5 h, leads to photosensitivity, during which birds regain the ability to respond to long day lengths in spring (Bentley, 2009).

Considerable attention has been given to the seasonal patterns in songbird species that sing exclusively during a certain season or to seasonal variations in song production and perception in species that sing year-round, along with the corresponding changes in their neural structures. Songbirds possess highly specialized neural circuitry for song production and perception, organized within two interconnected systems: the song control system and the auditory system. These systems exhibit remarkable plasticity, particularly in response to seasonal changes.

The song control system in songbirds consists of a set of discrete brain nuclei, including the HVC (proper name), the robust nucleus of the arcopallium (RA), and Area X as key components. The HVC integrates sensory inputs and coordinates motor outputs. The RA receives input from the HVC and projects to brainstem nuclei that control the vocal muscles and the syrinx. Area X, part of the basal ganglia, is involved in song learning and maintenance. Seasonal plasticity in the song control system is evident in many songbird species (for a review, see Rundstrom and Creanza, 2021). During the breeding season, these brain nuclei often undergo significant changes in size and neural connectivity. For instance, both the HVC and RA can increase in volume and strengthen their connection due to upregulation of neurogenesis and axonal sprouting. These changes are driven by variations in steroid hormone levels, particularly testosterone, which rise during the breeding season and enhance the birds’ singing behavior and song complexity (for a review, see Ball et al., 2004; Brenowitz, 2004).

The auditory system of songbirds is equally sophisticated, allowing them to discriminate fine temporal and spectral features of conspecific songs. Key components include the field L complex, analogous to the mammalian primary auditory cortex, and the caudomedial nidopallium (NCM) and caudomedial mesopallium (CMM), involved in higher-order auditory processing and memory (Woolley, 2012). During the breeding season and depending on the species, there can be increased responsiveness and selectivity towards conspecific songs or changes in neuronal preferences for specific song elements. Hormonal fluctuations play a pivotal role in modulating auditory sensitivity and neural plasticity. Aromatase, the enzyme that metabolizes testosterone and dihydrotestosterone into estrone and estradiol, is highly expressed in the NCM implicating the role of neuro-estrogens in sensory encoding and vocal communication (Vahaba and Remage-Healey, 2018). This, combined with seasonal changes in circulating testosterone, provides an elegant mechanism for seasonal adjustments in auditory perception or attention, as already demonstrated by our team in male starlings using functional Magnetic Resonance Imaging (fMRI) (Cousillas et al., 2013; De Groof et al., 2013, 2017; Van der Linden and Balthazart, 2018).

The seasonal plasticity and fine-tuning of the songbird’s song control and auditory systems underscore the adaptability of their neural circuits in response to environmental and hormonal cues. These changes are essential for optimizing song production and perception, which are vital for reproductive success and survival.

While most attention has been given to song production and perception in the context of male–female bonding and mating, or adult social bonding, particularly focusing on aspects of learned vocalizations that develop similarly to human speech (Bolhuis et al., 2010; Marler, 2004), the investigation of auditory responses to avian nestling calls extends far beyond the typical scope of songbird research. These “hunger” calls represent a nearly universal stimulus that elicits consistent behavioral responses across species, including humans, where such responses have been shown to depend on parental and associated hormonal status (for a review, see Witteman et al., 2019).

The current study marks the first attempt to explore whether begging calls elicit specific auditory responses in non-parenting songbirds, whether these responses are modulated by the hormonal status of adult birds as evoked by the artificially induced breeding conditions, and whether they reflect biparental care for offspring, focusing on the starling species.

Similar to pair formation in the breeding season, the introduction of nestlings constitutes a crucial step in a starling’s life. As an altricial species, European starling nestlings produce vocalizations at birth to solicit parental attention and care, such as food begging calls. Both parents participate in feeding the nestlings (Corney and Barber, 2018), although parental investment, especially in males, may vary considerably (Feare, 1984; Jimeno et al., 2014). The level of investment males make in feeding their young can range from equal to that of females to very minimal effort. For instance, bigynous males may contribute more to one brood than the other (Bruun et al., 1997). Although extra-pair paternity is possible, there is no evidence suggesting that males can distinguish between their own offspring and those of others, as they show similar levels of care in feeding both (García-Vigón et al., 2009). In all situations, there is clearly a sensitivity to chicks’ begging calls in both sexes.

Given that starlings are seasonally reproducing songbirds, exposure to nestling begging calls is expected only during the breeding season. Therefore, this study aims to investigate whether playbacks of these vocalizations in the absence of actual hatchlings evoke differential responses between the artificially induced breeding and non-breeding conditions, allowing birds to functionally classify these vocal signals based on their seasonal relevance. As starlings do not breed successfully in captivity, experiments or comparison with parenting birds is impossible.

The present study examines neural activations in adult male and female starlings that have not built nests, using auditory fMRI to compare responses to recorded nestling begging calls with responses to carefully selected adult starling song stimuli which served as controls. Auditory fMRI, a non-invasive imaging technique, enables visualization of differential neuronal responses to repeated presentation of stimuli in the same subject over time. Our previous auditory fMRI studies in male starlings have confirmed the seasonal relevance of specific vocalizations, reflected in neural activity within the caudomedial nidopallium (NCM), and demonstrated that the seasonal shift in auditory attention is mediated by local changes in estrogen (De Groof et al., 2013, 2017). Electrophysiological recordings further revealed seasonal plasticity in the preferences of field L auditory neurons in female starlings towards specific male song elements (Cousillas et al., 2013). In these previous studies the birds were exposed to starlings’ learned songs, classified into individual—and species-specific songs utilized both within and outside the breeding season by both sexes, albeit with varying abundance and relevance between seasons (Hausberger, 1997).

Specifically, the current study seeks to answer the following questions: (1) Is there a ‘universal’ response to begging calls even in non-parenting birds? (2) Which regions of the avian auditory forebrain perceive begging calls, and how does this activation compare to other social species-specific or artificial control stimuli? (3) Does this activation pattern change between the artificially induced breeding and non-breeding seasons? (4) Is the region and intensity of auditory neuronal activation the same for both sexes?

## Materials and methods

### Ethics statement

All procedures and animal handling were performed in accordance with the European guidelines for the care and use of laboratory animals (2010/63/EEC) and approved by the Committee on Animal Care and Use at the University of Antwerp, Belgium (ECD 2018–88). At the end of this non-invasive neuroimaging study, the birds were returned to the aviaries of the University of Antwerp. No animals were killed for the present study.

### Subjects

Eleven adult male and nine adult female European starlings (*Sturnus vulgaris*) (85 ± 10 grams) were used in this experiment. Birds were wild caught in Cyprus in January 2018. The population was divided into two mixed-sex groups and housed in two indoor aviaries sized 6.47 m^3^ (1.40 m x 2.20 m x 2.10 m) containing tree branches, of the Bio-Imaging Lab at the University of Antwerp (Antwerp, Belgium). No nest boxes were provided; hence no nesting behavior was observed. Food and water were available *ad libitum* in the aviaries.

### Photoperiodic manipulations to induce seasonality

**Figure 1:**
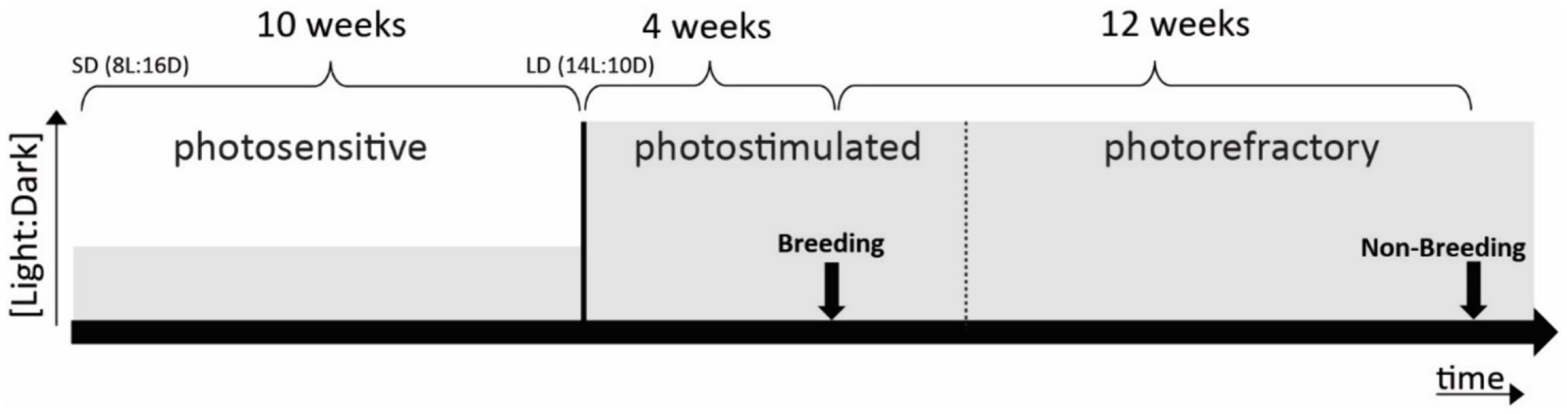
Schematic representation of photoperiodic manipulation to induce photostimulation and photorefractoriness. Arrows indicate fMRI scanning periods (one in breeding and one in non-breeding periods). Solid lines mark clear transitions between photostages, whereas the dotted line represents a gradual transition. Grey levels indicate the [light:dark] ratio.

To investigate whether begging calls are categorized differently based on seasonal behavioral relevance, we controlled seasonality by an artificial light–dark cycle. This is an approved method to induce photostimulation (breeding) and photorefractoriness (non-breeding) (Dawson et al., 2001) validated also by our group as shown by sex hormone measurements in starling plasma in previous studies (e.g., Orije et al., 2021). This regime consisted of 10 weeks of short days (SD: 8 h light) followed by 16 weeks of long days (LD: 14 h light), which successfully simulated natural photoperiodicity at an accelerated rate (Bernard and Ball, 1997; De Groof et al., 2013, 2017) (Figure 1).

Birds were made photosensitive in anticipation of the experiments by shifting them to SD (Figure 1). After 10 weeks of SD conditions, birds are photosensitive and ready to respond to LD stimulation. The subsequent shift to LD induces the seasonal development of their gonads and song control system plasticity to mimic the breeding season (photostimulated condition). After approximately 4 weeks of LD exposure, starlings reached peak photostimulation, characterized by yellow beaks and increased song performance. After another 12 weeks of LD starlings lost their sensitivity to long daylight periods (photorefractory condition) as indicated by post-nuptial feather molting, which occurs when gonads are regressing and plasma testosterone levels drop (Dawson, 2003; Gwinner, 1977).

The starling’s brain activity was visualized using auditory fMRI after 4 weeks of photostimulation (artificial breeding season) and after 12 weeks of photostimulation (artificial non-breeding period) (see Figure 1). All animal handling and experimental procedures were performed as outlined by De Groof et al. (2013, 2017).

### Anesthesia and physiology

In preparation for the MRI session, birds were individually retrieved from their home cage in a transportation box to reduce external auditory and visual stimulation and minimize confounding factors that could influence neural responsivity during the fMRI scans. Additionally, they were then kept in sensory-reduced conditions for at least 30 min before scanning.

Animals were anesthetized with a 0.2 mL intramuscular bolus injection in the pectoral muscle containing a mixture of medetomidine (10 mL, 1 mg/mL Domitor, Pfizer, Germany) and ketamine (0.5 mL, 50 mg/mL Anesketine, Eurovet Animal Health, the Netherlands). Anesthesia was maintained through continuous intramuscular infusion with the same anesthetic mixture at a rate of 0.12 mL/h. Minutes after injection, consciousness was assessed with the toe pinch test before the birds were placed in the MRI scanner. Each anesthetized animal was placed in a custom-built beak holder in a head-prone position. The head was secured in place with tape to prevent movement, without covering the animal’s ear coverts to not interfere with auditory stimulation. Possible wing movements were limited by wrapping a jacket around the torso of the animal (De Groof et al., 2013, 2017; Van Ruijssevelt et al., 2013).

During scanning, animals were breathing a mixture of oxygen and nitrogen (200 and 400cm^3^/min) delivered through the beak-holder. The breathing rate was followed with a pneumatic sensory pad underneath the animal’s chest (SA Instruments Inc., United States).

Body temperature was maintained at 40 ± 0.5°C using a cloacal temperature probe, connected to a feedback-controlled air heating system (SA Instruments Inc., United States). After scanning, birds were administered an intramuscular bolus of 0.2 mL Atipamezole (0.5 mg/mL Antisedan, Zoetis, United States) to reverse the effects of medetomidine.

### Auditory stimulation

**Figure 2:**
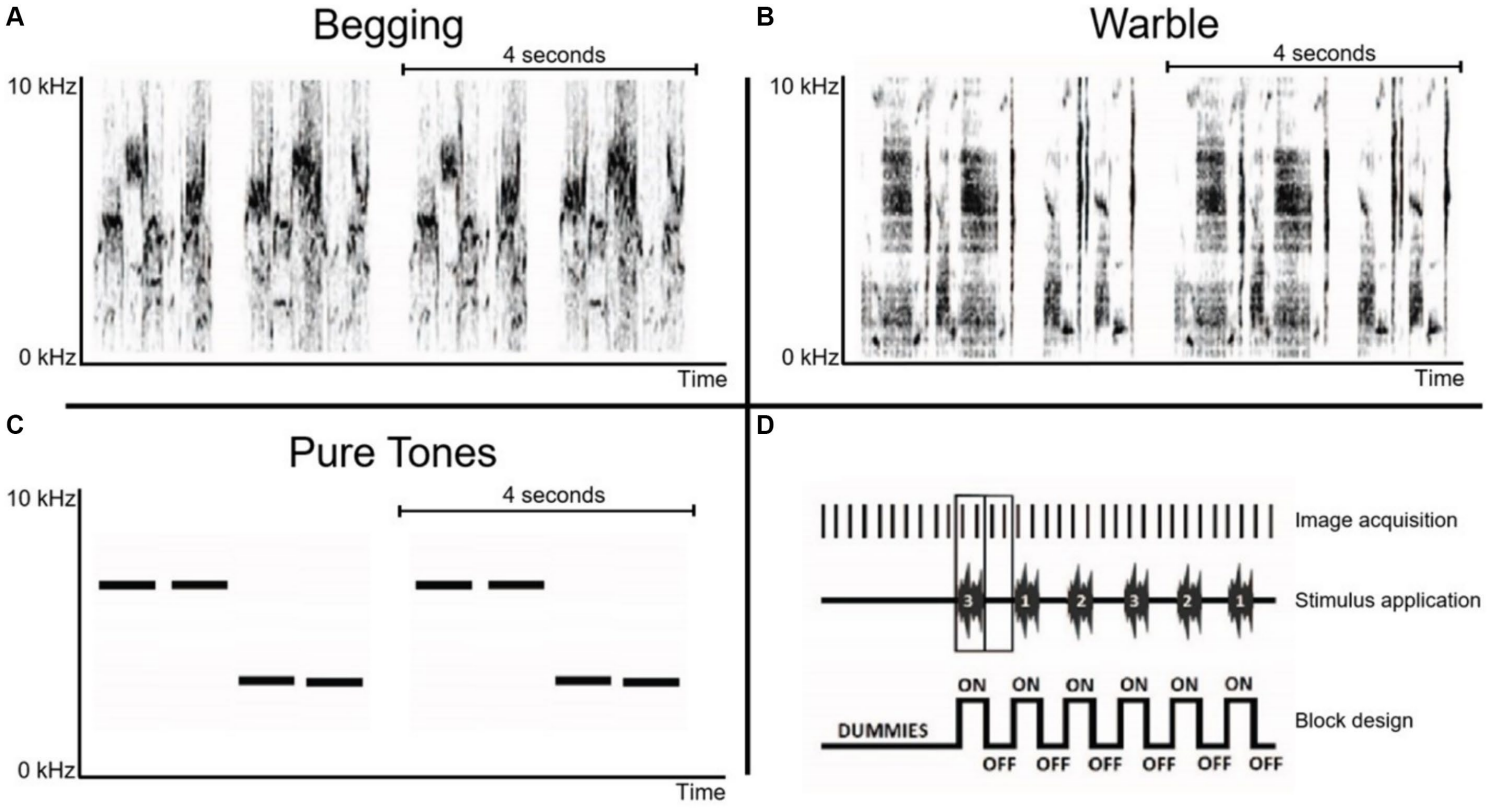
Overview of sonograms and acquisition paradigm. **(A)** Begging calls: monosyllabic vocalization that is loud and relatively long for a call and emitted in bouts directed at parents that are present at the nest (Elie and Theunissen, 2015). **(B)** Individual warbling motifs Class 3A: initial motifs taken from a quiet warbling song (De Groof et al., 2013). **(C)** Pure tones: 3 kHz and 7 kHz artificial control sounds. Horizontal lines in panels A–C indicate the sound patterns with a duration of 4 s, which are presented twice during the acquisition of a single fMRI image. **(D)** Schematic representation of the initial part of a randomized block design with each ON-block representing the acquisition of two BOLD fMRI images during auditory stimulation, interweaved by OFF-blocks consisting of two fMRI images acquired during rest (baseline). The stimulus presentation protocol was preceded by 10 dummy scans.

Magnetless dynamic speakers (Visaton, Germany) connected to a desktop featuring Presentation V18.3 (Neurobehavioral Systems Inc., United States) in the control room were used as stimulation device. Between different subjects’ scans, the left and right speakers were switched to account for any hemispheric bias in auditory stimulation resulting from potential speaker inequality. Song individual warbling motifs, see Hausberger (1997) and begging sequences were obtained from the Animal and Human Ethology research group of the University of Rennes (Rennes, France) and were unfamiliar to the starlings in this experiment. Warbling motifs are repeatable units, composed of several notes, within the long sequences of continuous song that are part of starlings’ song repertoire. Warbling sequences include three main types of motifs: individual-specific motifs in the first part of the song, species-specific clicks and the high-pitched trills (e.g., Adret-Hausberger and Jenkins, 1988). In the present study, Individual-specific warbling motifs (Class 3A) were selected as naturalistic control sound for two reasons: (1) they are important in social communication and (2) previous work has demonstrated that the response to this type of stimulation did not change between seasons (De Groof et al., 2013). These motifs were recorded from a single unfamiliar male starling (Figure 2B). Nestlings’ begging call bouts were recorded from within their nest (Figure 2A). There can be variations in acoustic structure between and within broods and according to the nestlings’ ages, sex and hunger state (Cotton et al., 1996; Reers and Jacot, 2011; Saino et al., 2008). Therefore, we used several naturalistic recordings performed in one nest while the whole brood was begging, at 5, 10, 15 and 18 days after hatching. The brood included 5 nestlings, 3 females and 2 males. We also made sure that there was no difference in the complexity of both species specific stimuli by measuring Shannon entropy using the specprop function in the R seawave package (sampling rate 22,050 Hz, window length 250 pts). The measures indicated 0.90 and 0.92 for the two begging call stimuli, and 0.89 and 0.92 for the two warbling motifs, which means that both stimuli did not differ in terms of complexity and that we could therefore ensure that potential differences in neural responsiveness could not be explained by sound complexity. Artificial pure tones (stimulus made of 3 kHz and 7 kHz, interleaved with 0.5 s of silence) were also included as a control to exclude potential seasonal changes in auditory acuity (Figure 2C). The intensities of the different stimuli were normalized to 67 decibels with Praat V6.0.50 software (Paul Boersma, University of Amsterdam).

The stimulus protocol consisted of a randomized block design of 16 s ON / 16 s OFF blocks. Each stimulation ON-block comprised 4 repeated sound patterns of 4 s with each pattern having two similar sound fragments presented in an AB pattern with 0.5 s rest between every sound pattern. This resulted in a total stimulus duration of 16 s per ON-block, followed by a complementary OFF-block of 16 s. Each stimulation block was presented 21 times per stimulus (begging calls, individual warbling motifs, pure tones), and two fMRI images (sampling rate of 8 s per image) were recorded per stimulation (ON or OFF) block, resulting in 252 MRI images. Ten dummy repetitions without stimulation were included at the start of the fMRI scan to allow for magnetization stabilization, giving a total of 262 MRI images per scan (Figure 2D).

### Image acquisition

All MRI measurements were performed on a PharmaScan 70/16 USR horizontal MR system (Bruker, Germany) equipped with a volume transmit coil and a four-channel parallel receive array coil (Bruker, Germany). To investigate changes in the Blood Oxygen Level Dependent (BOLD) signal over time, T2-weighted turbo-RARE (Rapid Acquisition Relaxation Enhanced) images were acquired with the following parameters: Effective TE = 50.60 ms, TR = 2000 ms, RARE factor = 8. Fifteen sagittal whole brain slices were recorded with a ventral-dorsal read orientation, a slice thickness of 1.0 mm, and a 0.08 mm slice gap. The matrix size was [64 × 32], with a field of view of (27 × 27) mm^2^ giving an in-plane resolution of (0.33 × 0.67) mm^2^. Additionally, fat suppression was enabled, and saturation slices were used to remove signals originating from adipose tissue in the neck and rapid eye movements. Functional image time series were reconstructed with a trapezoid filter (0.25 × 0.75) in the frequency and phase encoding direction.

An fMRI scan was considered successful if (1) a significant BOLD response could be detected in the auditory forebrain at the exploratory threshold P_uncorrected_ < 0.05, (2) framewise displacement was no more than two voxels and (3) no artifacts were present in the images. Unsuccessful scans were repeated after a minimum of 2 days, allowing birds to recuperate from anesthesia.

### Image processing

The raw datasets were acquired with ParaVision 6.0.1 (Bruker, Germany) and converted to the NifTI file format, using an in-house script in MATLAB R2017b (MathWorks, United States). Statistical Parametric Mapping 12 (SPM12) (FIL methods group, University College London) was used to align all functional images to the first image, based on a six-parameter (rigid body) spatial transformation. A mixed-sex population-based template was created from the first repetitions of each fMRI scan in Advanced Normalization Tools (ANTs). Next, fMRI scans of all subjects and all seasons were registered to this study-based template in SPM using a 12-parameter affine transformation, followed by non-linear deformations. Finally, the data were smoothed in-plane using a Gaussian kernel with an FWHM of (0.66 × 1.34) mm^2^. We applied a high pass filter (352 s) to remove low-frequency drift in the BOLD signal. Next, for each subject, the BOLD signal in each voxel was modeled with a Finite Impulse Response function. A starling MRI atlas (De Groof et al., 2016) was normalized to the study-based template, which functioned as a high-resolution anatomical reference. This atlas treats all nuclei as a single entity and cannot separate between functional subdivisions of the NCM and Field L. Hence, all conclusions will be based on approximations of delineations provided from the literature (Fortune and Margoliash, 1992; Pinaud et al., 2006).

### Statistical analysis

Statistical voxel-based analyses were performed for each scan using a mass-univariate approach based on the General Linear Model (GLM) implemented in SPM12. In each voxel, the significant BOLD response for the three stimuli was computed by digitally subtracting the OFF signal from the ON signal. *T*-contrasts (stimulus > rest) were defined for each stimulus separately and together, resulting in contrast files to be used in the next processing steps.

First, a one-sample *t*-test was performed on the first-level contrasts of all stimulations (begging calls, individual warble, and pure tones) over rest blocks. The voxels that demonstrated significant activity over rest were used as a region of interest for small-volume correction in further analysis. Next, separate ANOVA models were designed to model the separate effects of sex, season, and stimulus, and finally, a grand design containing all factors to model potential interaction effects in a three-way ANOVA was used.

The familywise error (FWE) correcting method using random field theory that is built-in SPM12 was used to correct for false positives. Only findings with a cluster-wise error rate of *P*_FWE_ < 0.05 that was larger than ten voxels (k*_e_* > 10) were considered statistically significant.

## Results

A longitudinal auditory fMRI study was conducted to visualize and quantify the neural responses evoked in male and female starlings when exposed to juvenile begging calls, which exhibit pronounced seasonal variations in relevance. To achieve this, starlings were successively placed under artificial breeding and non-breeding conditions, regulated by light regimes, without access to nest boxes or offspring to evaluate the influence of seasonality and sex on their auditory processing.

### Begging calls elicited stronger bilateral activation in the auditory forebrain of non-parenting birds compared to other social and artificial sounds, irrespective of season or the sex of the receiver

**Figure 3:**
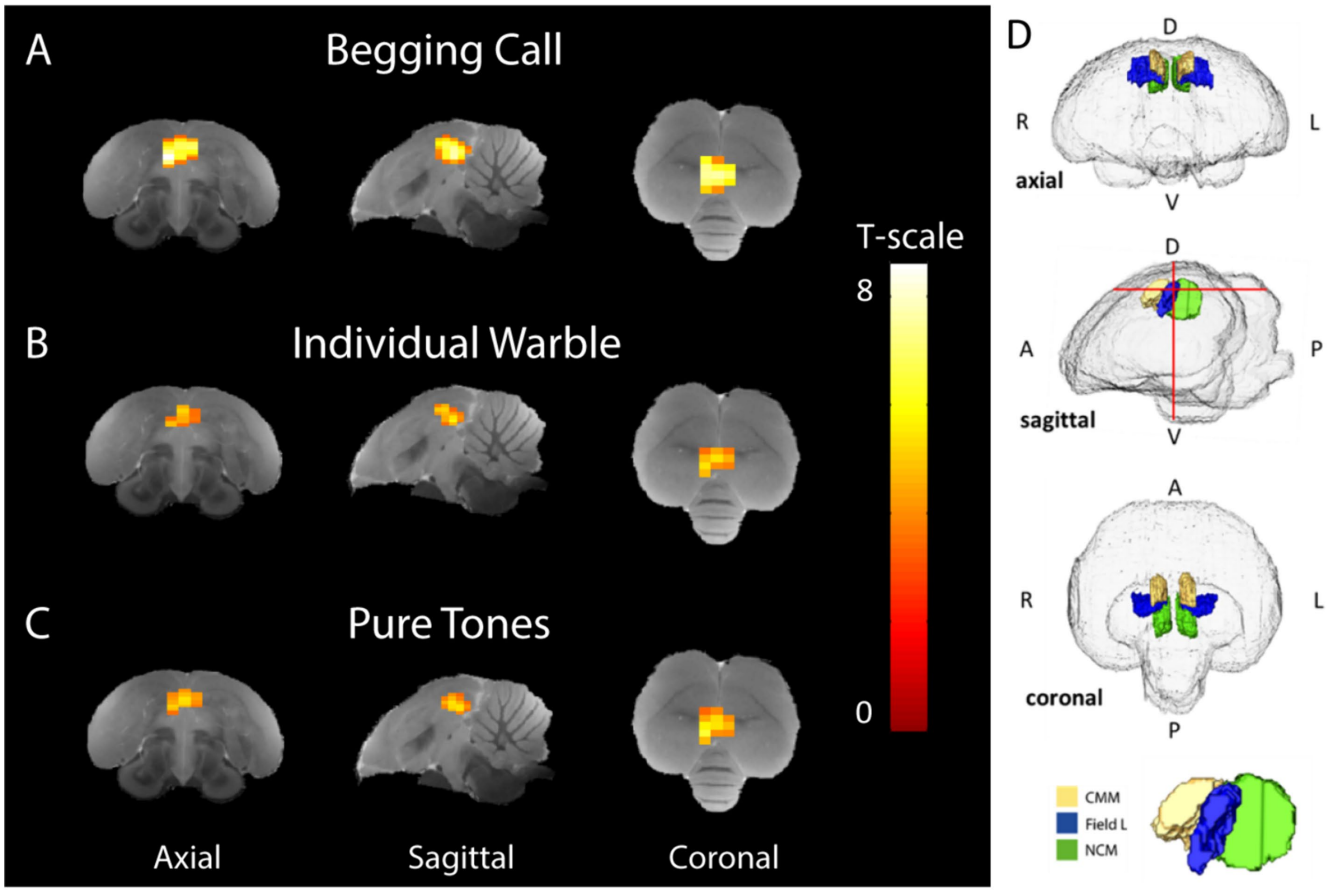
Average stimulus-specific neural activation (breeding and non-breeding season in both sexes) higher than rest periods. Voxels showing BOLD signal responses that are significantly stronger during stimulation than rest blocks have been superimposed on the starling MRI atlas (De Groof et al., 2016) for **(A)** begging calls, **(B)** individual warbles class 3A, and **(C)** pure tones (3 + 7 kHz). The color bar indicates the *t*-statistics for each voxel. **(D)** Transparent volume rendering of the avian brain containing color-coded volume renders of auditory forebrain structures with crosshairs indicating the slice orientation displayed in panel A-C and on the bottom, a 3D render with yellow, blue, and green indicating CMM, Field L, and NCM, respectively, (One-sample *t-tests*: P_Unc_ < 0.001, k_voxels_ > 10). D = dorsal, V = ventral A = anterior P = posterior, L = left, R = right.

Firstly, one sample *t*-tests were performed at the group level for each stimulus over baseline to confirm the responsiveness to all stimuli and to assess the topography and the relative response amplitude of the BOLD response to each stimulus, regardless of seasonality or sex. This revealed activations to all stimuli in Field L, NCM, and CMM (Supplementary Figure S1; P_UNC_ < 0.001, T_max_ = 9.24 and k_voxels_ = 69 voxels). This activation upon all stimuli over rest was used as a mask for small volume correction in the subsequent voxel-based statistics. Subsequently, one sample t-tests for each separate stimulus compared to the rest block demonstrated activation in the bilateral auditory nuclei Field L (primary auditory region), NCM, and CMM (secondary auditory regions) for every stimulus (Figures 3A–C).

Begging calls elicited more widespread BOLD activation compared to the warbles and pure tones, resulting in a larger cluster of 81 voxels in the auditory forebrain (P_FWE-peak_ < 0.0001, T_peak_ = 8.51) (Figure 3A). The individual warble motifs produced smaller spatial activation than the begging calls, resulting in a cluster of 45 voxels located in Field L, NCM, and CMM (P_FWE-peak_ = 0.002, T_peak_ = 5.83) (Figure 3B). Pure tones of 3 and 7 kHz, elicited activation that was spatially and statistically similar to the individual warbles with a significantly higher BOLD response compared to baseline in a total of 53 voxels in Field L, NCM, and CMM (P_FWE-peak_ < 0.0001, T_peak_ = 6.33) (Figure 3C). Overall, the begging calls activation is stronger and recruits a larger area in the auditory regions (Figure 3D), with higher *t*-statistics as compared to the naturalistic and artificial control sounds. Moreover, these responses were elicited in non-parenting male and female birds demonstrating a ‘universal’ response type elicited by offspring begging calls.

### Begging calls produce a differential activation in comparison to control sounds in right field L and NCM, with the highest difference in activation in right field L

**Figure 4:**
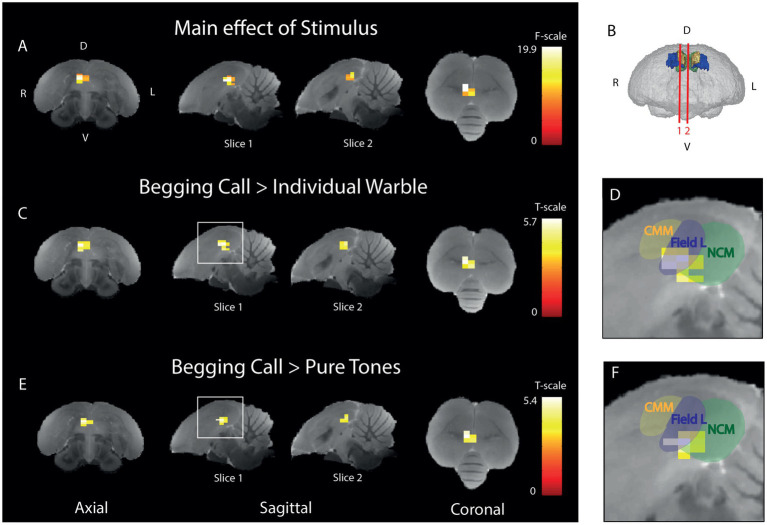
Begging calls sensitivity is highest in right ventral Field L and midsagittal ventral NCM **(A)** Three orthogonal views showing the significant main effect of stimulus of the BOLD response. The color bar indicates the *F*-statistics for each voxel. Only the right (R) hemisphere is depicted as no differential stimulus sensitivity was observed in the left (L) hemisphere **(B)** 3D surface rendering of the starling brain visualizing the slice orientations presented in panel A-F at the level of the auditory forebrain 1.4 mm from the midline (slice 1) and 0.16 mm from the midline (slice 2). The color bar indicates *t*-statistics for each voxel. **(C)** *Post-hoc* test of begging calls (both seasons) compared to individual warbles class 3A (both seasons). The color bar indicates the *t*-statistics for each voxel. **(D)** Zoom-in on peak activation of the significant cluster observed in the white square on slice 1 in panel C, overlaid with delineations of CMM, Field L, and NCM. **(E)** *Post-hoc* test for begging calls (both seasons) compared to pure tones (3 + 7 kHz) (both seasons). The color bar indicates the *t*-statistics for each voxel. **(F)** Zoom-in on the significant cluster observed in the white square on slice 1 in panel E, overlaid with delineations of CMM, Field L, and NCM. (ANOVA and *post-hoc* tests; P_FWE_ < 0.05, k_voxels_ > 10). D = dorsal, V = ventral, L = left, R = right.

To pinpoint which voxels have a differential activation upon begging calls, individual warble, and pure tones, an ANOVA was performed to test the main effect of stimulus (Figure 4A). This revealed a cluster of 16 voxels in right Field L and NCM (P_FWE-peak_ < 0.0001, F_peak_ = 19.89). *Post-hoc* *t*-tests were performed to test if the amplitude of the BOLD response was significantly higher for the begging calls compared to the other stimuli. Therefore, we statistically compared the relative signal increase over the baseline of the begging calls to the relative activation over the baseline for individual warbles (Figure 4C) and pure tones (Figure 4E). For the begging calls compared to individual warbles, we observed significantly higher activation for begging calls in Field L and NCM with the biggest difference in activation (white voxels in Figure 4) in Field L in a cluster of 19 voxels (P_FWE-peak_ < 0.0001, T_peak_ = 5.72). For the comparison with pure tones, a cluster of 14 voxels was also found in Field L (P_FWE-peak_ < 0.0001, T_peak_ = 5.41). No other pairwise comparison of stimuli revealed voxels that produce significant activation, indicating that, specifically the begging calls produce stronger activation than the other stimuli in Field L and NCM with the highest significance in Field L.

### Begging calls responsiveness is modulated according to seasonal relevance

**Figure 5:**
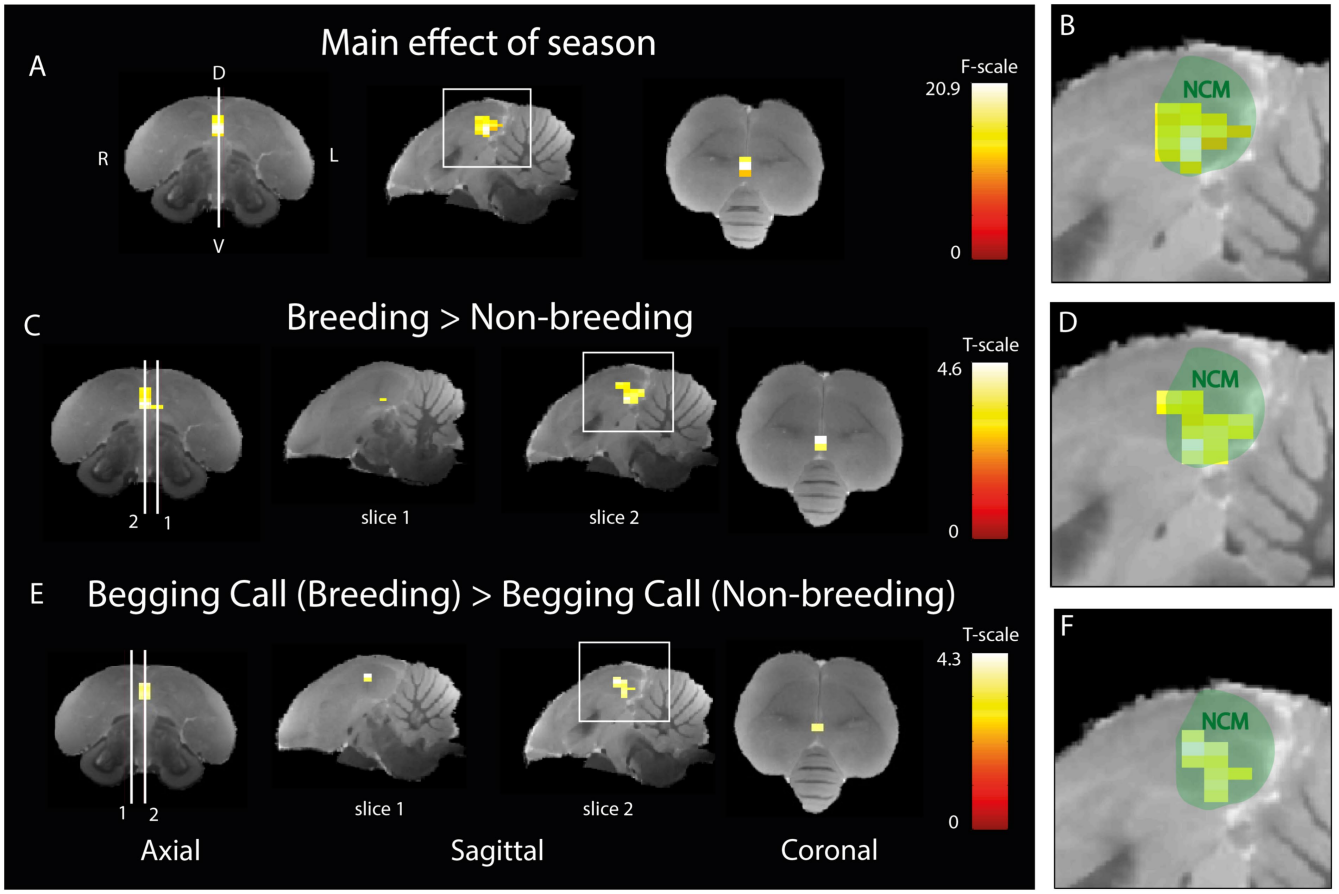
Seasonal difference in the processing of begging calls **(A)** Three orthogonal views illustrating the significant main effect of season (different BOLD responses over rest for breeding compared to non-breeding seasons for all stimuli together). The color bar indicates the *F*-statistics for each voxel. **(B)** Zoom-in on the area in the white square in sagittal view in panel A overlaid on a green delineation indicating NCM. **(C)** *Post-hoc* comparison of all stimuli during breeding versus non-breeding season. White lines 1 and 2 refer to the position of sagittal slice 1 (1.4 mm to the left from the midline) and 2 (0.16 mm to the right from the midline) respectively. The color bar indicates the *t*-statistics for each voxel **(D)** Zoom-in on area in the white square in sagittal view in panel **(C)** overlaid on a green delineation indicating NCM. **(E)** *Post-hoc* comparison of differential activation to begging calls stimulation in the breeding versus the non-breeding season. White lines 1 and 2 refer to the position of sagittal slice 1 (1.4 mm to the right from the midline) and 2 (0.16 mm to the right from the midline) respectively. The color bar indicates the *t*-statistics for each voxel (**F**) Zoom-in on the area in the white square in sagittal view in panel E overlaid on a green delineation indicating NCM. (ANOVA and *post-hoc* tests; P_FWE_ < 0.05, k_voxels_ > 10). D = dorsal, V = ventral, L = left, R = right.

The next aim was to investigate whether the activation in response to auditory stimulation changes between artificially induced breeding and non-breeding seasons. A two-way ANOVA was performed to investigate the main effect of season (Figures 5A,B) including both sexes and upon all types of auditory stimuli. A significant seasonal effect was found in ventral NCM covering a cluster of 15 voxels (P_FWE-peak_ = 0.001, F_peak_ = 20.86). *Post-hoc* *t*-tests revealed that the activation is higher in the breeding season in 18 voxels of NCM (P_FWE-peak_ = 0.001, T_peak_ = 4.57) compared to the non-breeding season (Figures 5C,D). No significant voxels were found that displayed a higher activation in the non-breeding season compared to the breeding season. Next, we investigated the seasonal differences in activation for the begging calls only by using a *t*-test including both sexes. A significant increase was found in response to the begging calls in the breeding season compared to the non-breeding season (Figures 5E,F; 11 voxels, P_FWE-peak_ = 0.002, T_peak_ = 4.26). The opposite contrast for begging calls in the non-breeding season compared to the breeding season did not result in any significantly different voxels. No significant seasonal differences were found when investigating the individual warbling motifs or the artificial pure tones confirming the findings of De Groof et al. (2013, 2017). Seasonal differences in response to begging calls are located ventrally in the midsagittal region of NCM.

### Sex does not influence the seasonal differential responsiveness to begging calls

Finally, we also investigated if the seasonally different activation for the begging calls in NCM and the intensity of the activation is the same in both sexes. While the two sexes did not respond differently to the begging calls by looking at the main effect of sex, taking together both seasons, the change in perception of the begging calls between seasons may be processed differently by males compared to females. To this end, we tested if there are any voxels where begging calls elicit higher BOLD responses compared to control sounds, in the breeding season vs. non-breeding season for males and females separately. This three-way interaction revealed no significant differences in activation patterns between males and females.

## Discussion

The present study reveals that begging calls elicit a universal auditory response in the right ventral Field L and midsagittal ventral NCM of non-parenting male and female starlings. This response is significantly stronger compared to other social and artificial control sounds. Notably, the highest differential activation, in contrast to control stimuli, is observed in the right Field L, indicating the heightened sensitivity of the primary auditory system to these universal acoustic signals crucial for offspring survival and fitness.

Furthermore, our data indicate that the response to begging calls is seasonally modulated, with the highest response occurring during the artificially induced breeding season. This seasonal variation in the response is observed in a ventral midsagittal portion of the NCM, a secondary auditory area, and remains consistent across both sexes. These findings underscore the bi-parental care for nestlings in starlings and provide evidence for the precise fine-tuning of offspring vocal communications between sender and receiver within a breeding context.

### Right side dominance of universal responses

The present study further highlights a robust engagement of the right hemisphere, particularly the right ventral Field L, in processing nestlings’ begging calls. This finding aligns with human research, where fMRI studies investigating adult responses to infants’ hunger cries have shown a similar right hemisphere dominance (Witteman et al., 2019). Lateralization of brain functions appears as a property of most or all vertebrates (e.g., Rogers and Andrew, 2002). On the basis of the known left dominance for speech processing in humans, Nottebohm (1971) investigated lateralization of bird song and found evidence for left hemispheric dominance for song production. However, on the perceptual side, like humans who process the verbal content in the left hemisphere and the emotional content (prosody) in the right hemisphere (Broca, 1861; Wernicke, 1874), songbirds show a functional distribution between hemispheres when processing different types of sounds, in particular in Field L, the primary auditory area (Cynx et al., 1992; George et al., 2004, 2005). It has been proposed that lateralization of brain processing of natural sounds would increase computational speed and avoid possible conflicting computation by the other side of the brain (Andrew, 1991; George et al., 2002). More precisely, the right hemisphere is hypothesized to process stimuli that require fine discriminations as well as familiarity or novelty (Andrew, 1974; Rogers and Andrew, 2002; Siniscalchi et al., 2008) while the left hemisphere would process stimuli with neutral or positive valence (e.g., Böye et al., 2005). Indeed, the involvement of the right hemisphere in processing arousal and survival signals has long been posited by the “right-hemisphere theory” (Rogers and Vallortigara, 2015), though recent evidence suggests it may also or rather be linked to the attention-grabbing nature of these “cries for help” (Andrew and Watkins, 2002; Hausberger et al., 2019). It has been proposed that lateralized processes concern more intensity/arousal than valence of stimuli (Baciadonna et al., 2018) and electrophysiological studies have shown a higher implication of the right hemisphere in attentional processes in humans (e.g., Ishii et al., 2014) and horses (Rochais et al., 2018). Indeed, the primary aim of begging calls is to elicit attention and prompt action in parents. In the present case, adults were anesthetized and hence did not have access to the usual additional visual stimulation of chicks begging with their open bills. Maybe the absence of visual information further enhanced the impact of the sole auditory information.

Moreover, it is particularly noteworthy that such heightened responses to juvenile calls are observed even in non-parenting birds. Responses to offspring distress calls appear to be a universal trait across all vertebrates exhibiting parental care and may extend to calls of other species’ young as well (Kelly and Schmidt, 2017; Root-Gutteridge et al., 2021), albeit with potential variations in the parameters governing auditory responsiveness (Massenet et al., 2022; Thévenet et al., 2023). In humans, even individuals without parental experience respond to infant cries, suggesting a general auditory proficiency that could contribute to caregiving adaptability (Bouchet et al., 2020).

### Photoperiodic modulation of hormonal state affects auditory processing of begging calls

The strictly regulated photoperiodic dynamics employed in this study to simulate natural seasonality serves as the primary driver for reproduction-related hormonal periodicity in seasonal songbirds (Nicholls et al., 1988). These light-induced hormonal changes largely account for the demonstrated seasonal differences in how these calls are perceived and interpreted. Research has linked exogenous testosterone treatment to enhanced nestling begging display and increased paternal feeding behavior (Boncoraglio et al., 2006; Noguera et al., 2013). While factors such as social structure, group dynamics, or nest box presence can also influence hormonal states and sexually dimorphic behaviors (Henry et al., 2013), they were not variables in this study. Additionally, non-hormonal factors like motivation or attention were excluded as birds were mildly anesthetized during fMRI scans. Together, these findings from the current and previous fMRI studies underscore the necessity for accurate neuromodulation in auditory brain regions under photoperiod-induced breeding conditions to effectively adapt to communication needs, be it male–male, male–female, or young-parent communication (Cousillas et al., 2013; De Groof et al., 2013, 2017).

Furthermore, in a previous study using Diffusion Tensor Magnetic Resonance Imaging, we demonstrated that the caudal NCM undergoes microstructural and volumetric changes during the breeding season (De Groof et al., 2009). This region exhibits expression of estrogen receptors and aromatase, as observed in other songbirds like zebra finches, white-throated sparrows, and canaries (Metzdorf et al., 1999; Saldanha et al., 2000; Pinaud et al., 2006; Apfelbeck et al., 2013; De Groof et al., 2017; de Bournonville et al., 2021; for a review, see Spool et al., 2022). Such expression creates a dynamic environment for spatiotemporal hormone fluctuations, facilitating estrogen-dependent modulation of auditory perception. These hormone-driven changes likely underlie the observed seasonal microstructural variations in the caudal NCM of starlings.

Notably, neuroendocrine modulation of human adult responses to infants’ hunger cries is a well-established phenomenon. Furthermore, studies have shown that hormonal modulation via exogenous testosterone administration can influence the processing of infant hunger vocalizations in women (Bos et al., 2010; Seifritz et al., 2003). This study in starlings provides evidence that the hormonal modulation of auditory responsiveness to enhance the perception of infant vocalizations may be an evolutionarily conserved trait not restricted to mammalian evolution.

### A shared differential processing of learned songs and conspecific calls in NCM

The transcription factor ZENK is an immediate early gene which functions as a surrogate marker of neuronal activity (Nordmann et al., 2020). ZENK expression studies suggest that neural activation differs between hearing learned songs and adult calls in songbirds, with birds showing a preference for conspecific vocalizations over pure tones (Phillmore et al., 2003; Pinaud and Terleph, 2008; Poirier et al., 2009). Begging calls in various avian species possess acoustic properties that reflect the nestling’s condition and convey species-specific information (Anderson et al., 2010; Ursino et al., 2018; Villain et al., 2015). Processing such complex acoustic characteristics occurs at the level of Field L, as demonstrated in European starlings in previous studies (Cousillas et al., 2004, 2013; George et al., 2003; Hausberger et al., 2000) and confirmed in the present study.

Categorization based on vocalizations’ social functions occurs more prominently in secondary auditory regions such as the NCM and CMM (Gentner and Margoliash, 2003; George et al., 2008). Our study demonstrates that both the right Field L and right/midsagittal NCM exhibit stronger responses to begging calls compared to other social and artificial sounds. However, while Field L neurons can adjust responses to songs based on the season, we observed seasonal changes in response to begging calls only in the midsagittal ventral NCM. In our previous fMRI research, we showed that the right caudal NCM also undergoes seasonal changes in response to learned conspecific vocalizations based on their behavioral relevance category in a particular season (De Groof et al., 2013, 2017).

### The role of sex in auditory perception of begging calls

Although both parents are involved in parental care in starlings, there is a high flexibility in the relative investment by each sex (Feare, 1984). Factors such as monogamous or polygynous status affects how primary or secondary females will receive help from the male (Bruun et al., 1997). In spotless starlings (*Sturnus unicolor*), a congeneric species, females increase their food provisioning rate in response to artificially increased nestling begging calls, suggesting a higher investment of females (Jimeno et al., 2014; Kolliker et al., 2000). One could expect in the current study also a sexual difference in the neural substrate responsible for this behavior, but this was not the case here. It may be that male–female differences would arise if actual parents (i.e., breeding birds) were tested, as shown for women involved in parental care compared to men (De Pisapia et al., 2013). This experiment would however be very difficult to perform, because starlings do not breed successfully in captivity. This is in direct contrast to mammals in which maternal lactation is the primary source of nutrition. It is therefore not surprising that in humans, gender-specific differences have been reported at the level of the amygdala and the orbitofrontal cortex when exposed to infant cry (Seifritz et al., 2003; Swain, 2008).

## Conclusion

fRMI recordings of adult brain responses in male and female starlings to the playback of nestlings’ begging calls suggest a deeply ingrained evolutionary foundation for the mechanisms governing the universal response of adults to the cries of hungry infants. The pronounced receptiveness to offspring’s begging calls among non-parenting individuals, evident in specific brain regions such as the secondary auditory cortex and the preferential right hemisphere, alongside hormonal neuromodulation, signifies a convergence in evolutionary adaptations aimed at facilitating adaptability and swift adult responses to these calls critical for offspring survival.

While variations in the intricacy of this system exist between lactating and non-lactating species, and the influence of human culture may account for the much simpler network observed here, the shared fundamental features underscore a compelling connection between avian and mammalian evolution. This evidence serves to bridge the evolutionary gap, highlighting commonalities in the mechanisms governing parental care across diverse species.

Functional neuroimaging studies in birds currently represent a very small percentage of the total number of non-human functional neuroimaging works, especially compared to those conducted on rodents and non-human primates (Mandino et al., 2020). Hopefully, our work on starlings may provide an example of an experimental paradigm that promotes the broader application of neuroimaging techniques to a wider range of bird species. Indeed, a better *in vivo* characterization of the neural underpinnings of fundamental biological functions, such as parental behavior, in birds may increase interest in avian neuroscience and lead to greater consideration of non-traditional model organisms.

## Data Availability

The datasets presented in this study can be found in online repositories. The names of the repository/repositories and accession number(s) can be found here: https://data.mendeley.com/datasets/r67bzxhxn5/2.

## Supplementary material

SUPPLEMENTARY FIGURE S1 One-sample *t*-test demonstrating significant (P_UNC_<0.001, k_voxels_ > 10) activation of average auditory stimulation block (begging calls, individual warble, and pure tones) over rest periods. Color bars indicate a significant BOLD-response higher than rest periods.
