# Supplementary figures and images for: Adult auditory brain responses to nestling begging calls in seasonal songbirds: an fMRI study in non-parenting male and female starlings (Sturnus vulgaris)
Source: Front Behav Neurosci. 2024 Sep 17;18:1418577. doi: 10.3389/fnbeh.2024.1418577 (PMC11442251; doi:10.3389/fnbeh.2024.1418577)

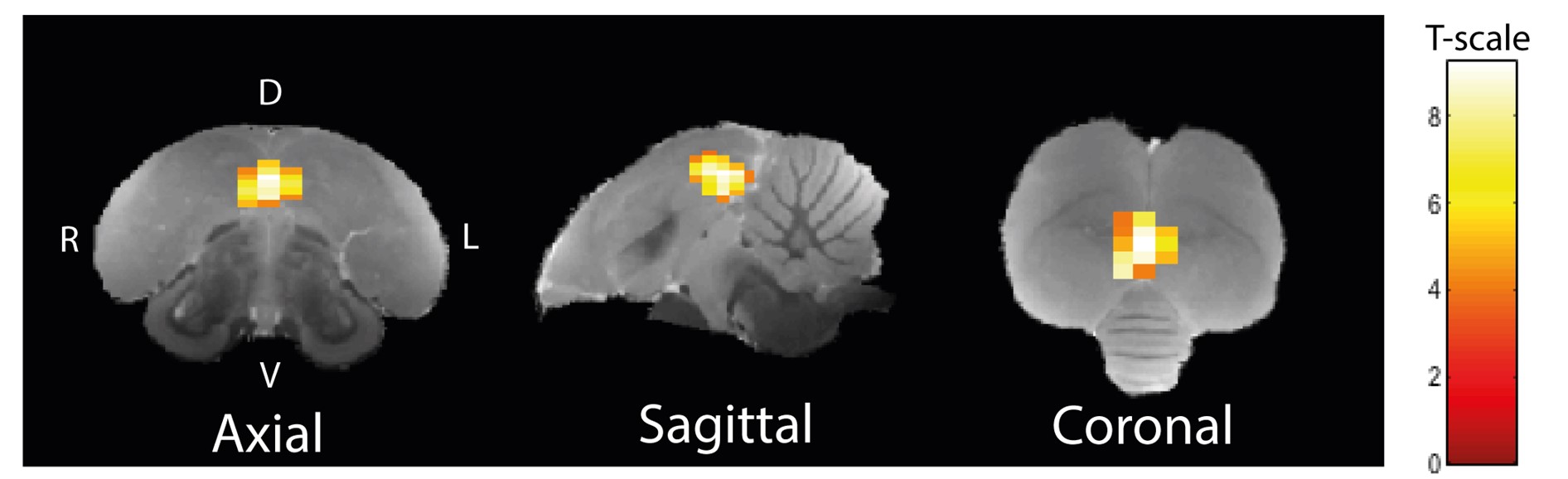

Supplement: SUPPLEMENTARY FIGURE S1 — One-sample t-test demonstrating significant (PUNC<0.001, kvoxels > 10) activation of average auditory stimulation block (begging calls, individual warble, and pure tones) over rest periods. Color bars indicate a significant BOLD-response higher than rest periods. [file Image_1.JPEG]
